# Miofrenuloplasty for Full Functional Tongue Release in Ankyloglossia in Adults and Adolescents—Preliminary Report and Step-by-Step Technique Showcase

**DOI:** 10.3390/medicina57080848

**Published:** 2021-08-20

**Authors:** Jakub Bargiel, Michał Gontarz, Krzysztof Gąsiorowski, Tomasz Marecik, Paweł Szczurowski, Jan Zapała, Grażyna Wyszyńska-Pawelec

**Affiliations:** Department of Cranio-Maxillofacial Surgery, Jagiellonian University Medical College, 30-688 Cracow, Poland; michal.gontarz@uj.edu.pl (M.G.); krzysztof.gasiorowski@uj.edu.pl (K.G.); tomasz.marecik@uj.edu.pl (T.M.); pawel.szczurowski@uj.edu.pl (P.S.); jan.zapala@uj.edu.pl (J.Z.); grazyna.wyszynska-pawelec@uj.edu.pl (G.W.-P.)

**Keywords:** miofrenuloplasty, ankyloglossia, tongue-tie, speech, frenotomy, tongue, frenectomy

## Abstract

*Background and Objectives*: Ankyloglossia is a functional term describing limitations of motor activity of the tongue due to the embryological malformation of the lingual frenulum. The lingual frenulum has a complex, three-dimensional structure, it is not only a mucosal fold, which connects the ventral surface of the tongue and the floor of the mouth. Such knowledge forced us to develop more advanced techniques for tongue release in ankyloglossia. The aim of this study is to describe a novel, precise surgical technique for tongue release. *Materials and Methods*: Miofrenuloplasty was performed in six patients with impaired tongue movements due to anatomical limitations. All of them were prepared for surgery and evaluated after the procedure by a speech therapist. *Results:* The healing process was uneventful in all patients. We did not observe any major complications. Tongue mobility and neck muscle tension improved significantly in all cases. In one case, the speech improvement was minor. *Conclusions*: Miofrenuloplasty is an advanced, but effective and highly predictable procedure for full functional tongue release in cases caused by MFGG complex. It should be done by experienced surgeon.

## 1. Introduction

The lingual frenulum is a tiny structure underneath the tongue that has the potential function of maintaining stability between the internal and external tongue muscles and, in some cases, when the structure is incorrect, may have a major impact on tongue movements. In anatomy textbooks, it is defined only as a distinct fold of the mucous membrane. Recent studies have shown that the lingual frenulum has a more complex anatomy, which can vary from individual to individual [1,2]. Similar observations can be seen in vivo during tongue-release surgery.

**Figure 1:**
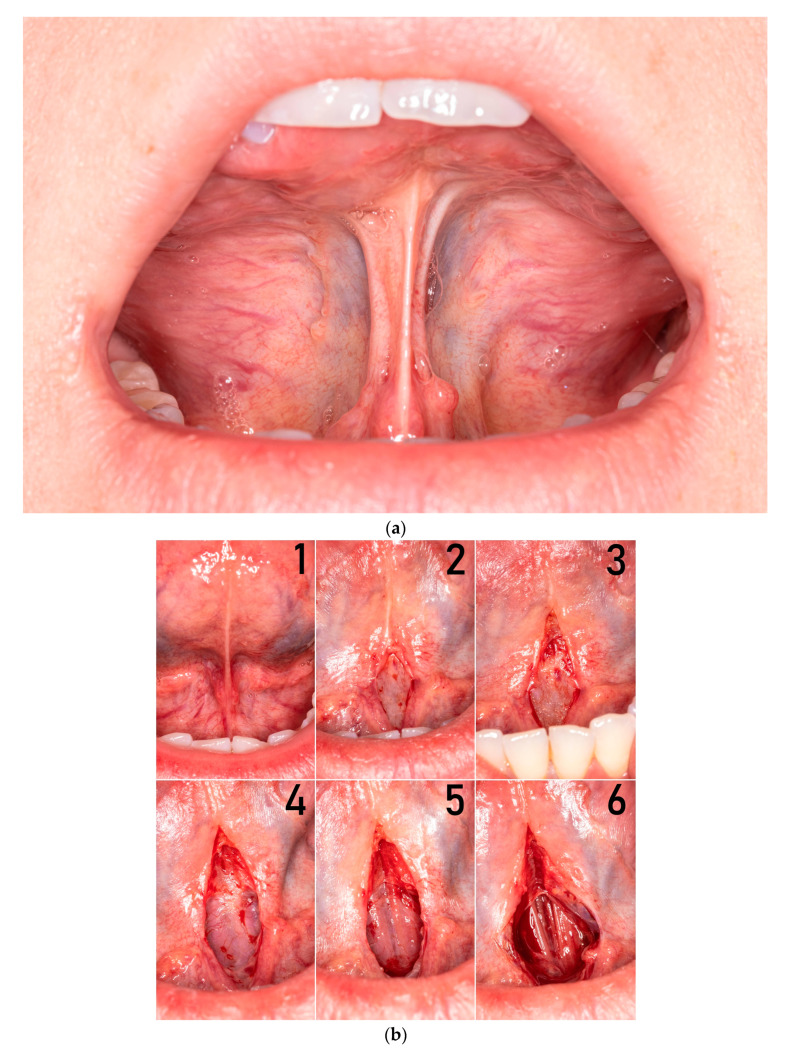
(**a**) In vivo anatomy of lingual frenulum (MFGG complex)**;** during LPS, only the anterior tongue is elevated. (**b**) In vivo anatomy of lingual frenulum (MFGG complex); during surgery, every layer is exposed. 1—elastic mucous membrane, 2—superficial fascia layer after frenotomy, 3—superficial fascia layer after frenectomy, 4—deep fascia layer after frenectomy, 5—perimysium of genioglossus muscle, and 6—anterior fibers of genioglossus muscle.

Ankyloglossia is a condition in which the motor functions of the tongue are impaired due to embryological malformation of the lingual frenulum and scarring after surgical interventions or trauma. The most common cause of ankyloglossia is tongue-tie syndrome, and the overall prevalence is 8% [3]. In some cases, restriction of the tongue may be caused by a short mucosal fold, sometimes by submucosal fascial fibers, and in some cases by the whole complex of mucosa, fascia, and anterior genioglossus muscle fibers (MFGG complex) (Figure 1a).

Undisturbed motor functions of the tongue are required for proper swallowing, chewing, breathing, and the development of speech. Together, these functions of the tongue are essential for the development of maxillofacial complex and have an impact on the quality of life.

There are multiple and various methods to treat tongue-ties: frenotomy, frenulectomy, frenuloplasty, Z-plasty, and V-Y plasty [4]. Most often, a simple frenotomy is performed with scissors or frenectomy with laser, both with various outcomes. Sometimes the same procedure is performed several times on the same patient. We believe that fibrosis and uncontrolled scarring result from an incorrect diagnosis and wrong choice of surgical technique.

There are no publications describing step-by-step surgical techniques to address these concerns. The aim of this study is to describe a novel, precise surgical technique of functional miofrenuloplasty for releasing the tongue in ankyloglossia caused by the whole MFGG complex and to evaluate the outcome.

## 2. Materials and Methods

Between January 2020 and March 2020, 6 adult patients (3 female, 3 male, age 18–37) with ankyloglossia were operated on using the miofrenuloplasty technique by the same surgeon in the Department of Cranio-Maxillofacial Surgery of the Jagiellonian University in Krakow. All patients were prepared by a speech therapist following the principles of myofunctional therapy to strengthen the internal and external tongue muscles and to train the lingual palatal suction (LPS), which is crucial for post-operative care.

To evaluate results, we used tongue range motion ratio (TRMR) with patient in the natural head position. The lingual palatal suction hold (TRMR-LPS) corresponds to the posterior tongue mobility, whereas the tip of the tongue in relation to the incisive papilla (TRMR-TIP) corresponds to the anterior tongue mobility [5]. Those functional measurements were evaluated before and 6 months after surgery. Other symptoms like snoring (Sn), suprahyoid neck muscle tension during tongue exercises (N), and speech impairment (Sp) were also taken into consideration.

Patients’ characteristics are presented in Table 1.

### 2.1. Functional Frenulum Anatomy

To improve the mobility of the tongue, it is mandatory to understand the three-dimensional dynamic structure of the frenulum. In vivo, the lingual frenulum consists of two to three layers (Figure 1b). The outer layer is elastic mucous membrane and the second layer is fascia, which differs in thickness flexibility and stretchability, most likely due to the histological structure (the ratio of type I collagen to type III collagen) [6]. The innermost, inconstant layer contains the anterior fibers of the genioglossus muscle and determines the MFGG complex.

Important anatomical structures that can be compromised during miofrenuloplasty are the lingual nerve, the deep lingual veins, sublingual duct, and caruncle.

### 2.2. Surgical Technique

All patients signed the informed consent prior to the treatment. Surgery was performed under local anesthesia with vasoconstrictor. The following instruments was needed for surgery: blade 15C, scalpel handle no. 3, Adson surgical tweezer, two mosquito forceps curved and straight, sharp Iris scissors, and a needle holder. The mean time of the procedure was 20 min. In all cases, polyfilament resorbable 5–0 sutures were used. Most of the procedure was done by blunt dissection. There was no need to use the electrocautery.

#### 2.2.1. Preparation

The patient’s collaboration is crucial before, during the procedure, and afterwards to ensure proper and predictable results. Sometimes it takes several months to prepare oral tissues and muscles for surgery. The patient elevates the tongue and performs LPS during every stage of the operation, which enables assessment of improved range of motion.

#### 2.2.2. First Stage (Exposing the Genioglossus Muscle)

**Figure 2:**
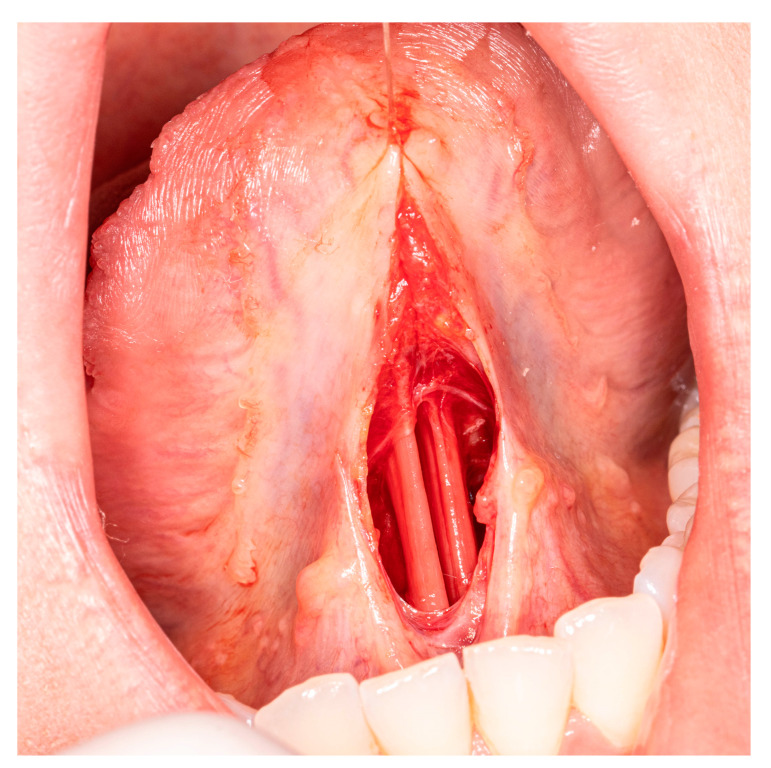
Anterior genioglossus muscle fibers limiting the tongue elevation.

After a horizontal incision is made with a 15C blade in the middle of the stretched frenulum, the tongue is lifted to gently open the wound in vertical dimension to visualize layer of fascia. A small V-shape incision is made to perform superficial frenulectomy. Care should be taken not to damage the venous branches and the lingual nerves, as they run close to the midline. Another horizontal incision is made through the second layer and blunt dissection is performed through epimysium to reveal the anterior fibers of the genioglossus muscle. It is recommended to put one traction suture in the upper aspect of the wound to stretch the muscles (Figure 2).

#### 2.2.3. Second Stage (Anterior Genioglossus Muscle Fibers Release)

**Figure 3:**
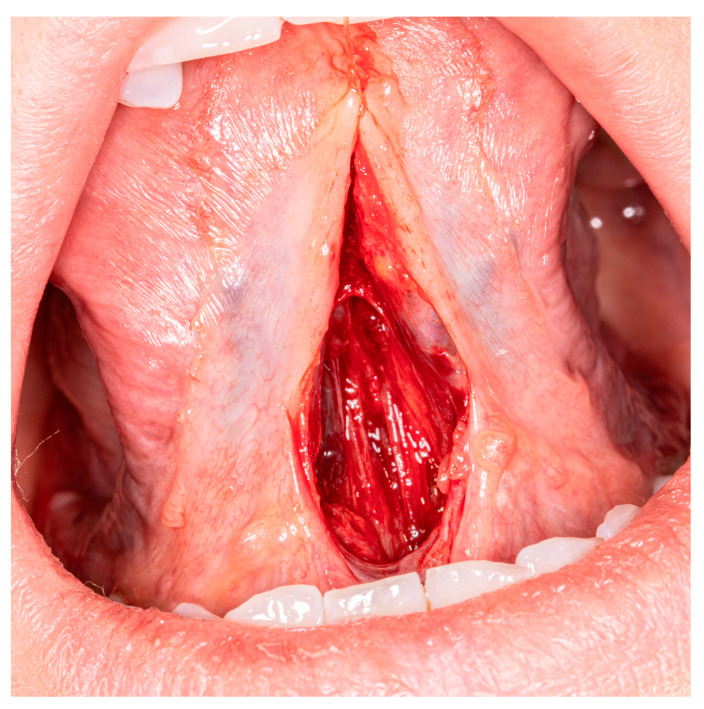
Fully released anterior fibers of genioglossus muscle.

During second stage, curved mosquito forceps are used very gently to divide the muscle fibers. In most cases it is not necessary to cut the muscle. During tongue elevation, these anterior muscle fibers are brittle and release spontaneously by themselves. The preparation is completed when the tongue is fully mobile or when the muscle fibers are less susceptible to separation (Figure 3). At this stage, the patient very often informs about an unpleasant pulling sensation in the suprahyoid region.

#### 2.2.4. Third Stage (Suturing)

**Figure 4:**
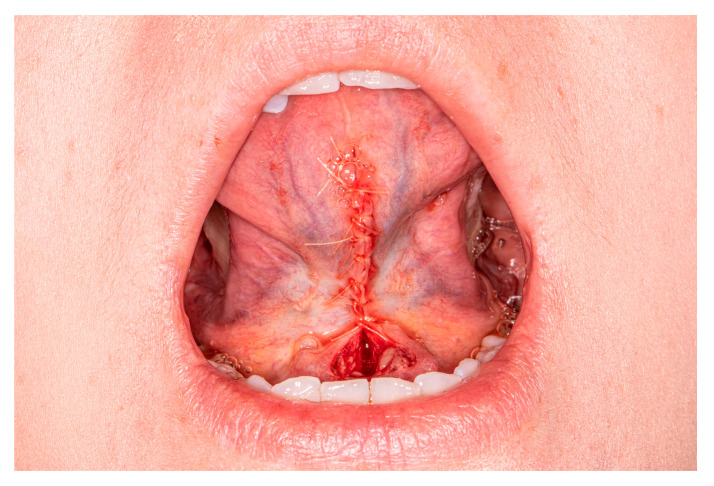
Final stage of the operation.

Suturing starts from the apex. Simple interrupted or vertical mattress sutures control tension. Sutures should be placed no closer than 2 mm to 3 mm from the flap edges to prevent tearing through the margins of the wound during postoperative swelling. During this stage, care should be taken not to damage the deep lingual veins and the lingual nerves. The wound in the lower part, close to the sublingual carancule, should be left opened for secondary healing (Figure 4). This prevents accidental closure of the sublingual ducts, generates less tension, and leaves a scar without contraction.

#### 2.2.5. Exercises in the Follow Up

**Figure 5:**
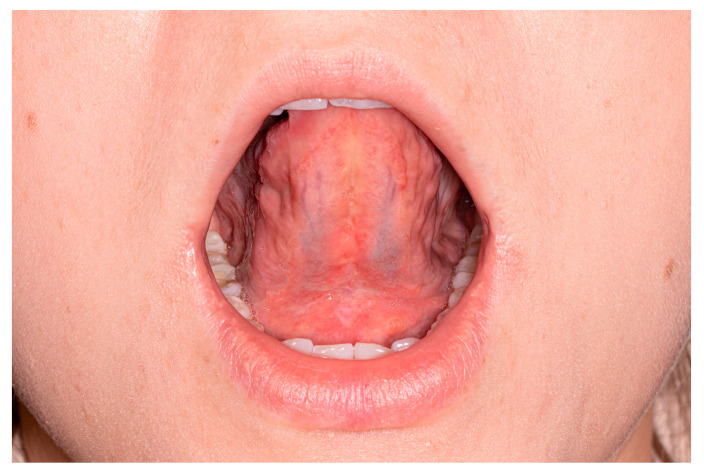
Final result after 1 month (TRMR-TIP 100%).

**Figure 6:**
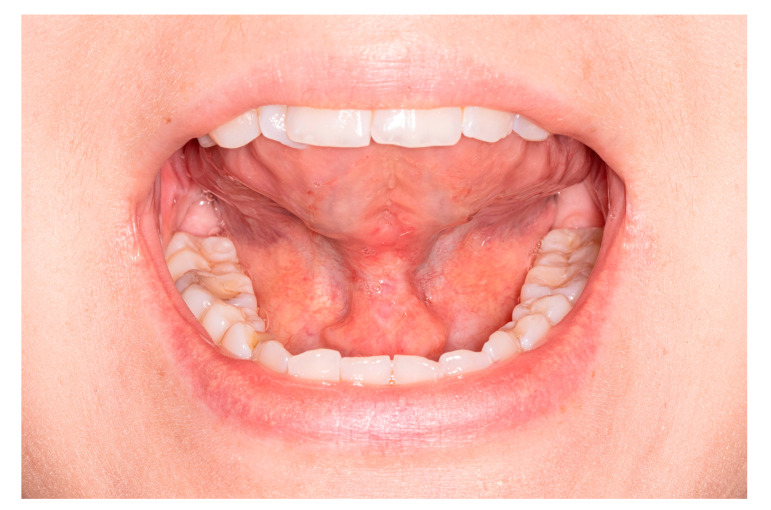
Final result after 1 month; the whole tongue reaches palate during LPS (TRMR-LPS > 60%).

On the first day following surgery, the patient starts exercises of the tongue. The patient performs exercises in front of the mirror, four times a day for 15 min, for two weeks to control the symmetry of lateralization and mouth opening with LPS and the tongue tip to incisive papilla. After 1–2 weeks, when the wound is healed, myofunctional therapy is implemented to make the scar elastic (Figure 5 and Figure 6).

## 3. Results

The healing process was uneventful in all cases with no major complications. Every patient experienced bruising of the ventral surface of the tongue and floor of the mouth for 3 to 7 days. Non-opioid painkillers were administered up to 72 h following the surgery. We did not observe loss of sensation and excessive fibrosis affecting the function. Tongue mobility, swallowing, and natural tongue position improved significantly. Proper and symmetrical tongue mobility was achieved in all cases without painful suprahyoid neck muscle compensations. After elastic scar formation lasting from 1 to 3 months, Grade I of the TRMR-LPS and TRMR-TIP motion parameters was achieved. In two out of four patients, the quality of speech improved. In one patient who reported snoring, it was not present after the procedure. The mean follow-up was 9 months. The results of treatment are presented in Table 2.

## 4. Discussion

Ankyloglossia is common entity, but there are no clear guidelines to its management for adult patients and adolescents. In symptomatic cases, some authors advocate for simple procedures such as frenotomy and some of them recommend more sophisticated ones [7,8]. Most of the typical tongue-ties cases are managed by frenotomy during the first year of life due to breastfeeding difficulties [9,10]. Despite the problem of this congenital disease, many different specialties have different opinions about the need of tongue release procedures [11].

The results of this study clearly demonstrated major improvements in tongue movement, speech, and neck muscle tension in moderate to severe ankyloglossia cases. In one patient, this procedure additionally eliminated snoring. One in four patients had only a minor improvement in speech. There were no complications during the surgery and healing process. All of the patients were evaluated by the same surgeon and speech therapist.

Selection of a surgical technique should be based on the structure of the frenulum, symptoms, corresponding compensations, and most importantly, motor function impairment and the age of the patient. In infants before the age of 6 months and later when the lingual frenulum is still fine, a cellular membrane frenotomy is recommended. In older patients, wherever possible, we should promote optimal healing by first intention. Establishing non-tension primary wound closure with soft polyfilament resorbable sutures is paramount for optimal postsurgical wound healing and scar formation. Frenectomy, frenoplasty, or miofrenuloplasty should be performed. Surgical skill should not influence the choice of a technique.

Recent anatomy papers define the lingual frenulum as a three-dimensional structure, not a midline mucosal fold or submucosal band. In vivo, during tongue-tie surgery, we observed that the structure of the lingual frenulum is multilayered. Most superficial layers composed of mucous membrane and fascia fibers are often not only structures limiting the mobility of the tongue. The choice of a surgical technique depends on which layer restricts the mobility of the tongue. The decision is made after clinical evaluation and thorough analysis of frenulum structure.

Miofrenuloplasty should be performed by an experienced oral or maxillofacial surgeon if the structure of the anterior fibers of genioglossus muscle limits the tongue mobility (MFGG complex). The second very important factor is the proper myofunctional preparation of the patient and postoperative care. Prior to surgery, the patient should be prepared by speech therapist. There are many methods to achieve this goal. In our practice the most important exercises are as follows: lateral tongue movements with controlling lower jaw deviation, tongue tip elevation, and lingual to palatal suction during wide mouth opening. Our goal is to stretch the muscles and prepare for the post-operative period. The average preparation time cannot be estimated. Each patient requires an individual approach.

## 5. Conclusions

In conclusion, the miofrenuloplasty is an advanced, but effective and highly predictable procedure for full functional tongue release in the hands of an experienced surgeon. The indication for this technique is ankyloglossia caused by the MFGG complex. The condition for obtaining a predictable result is cooperation with the patient and the speech therapist.

## Figures and Tables

**Table 1:** Characteristics of patients before the surgery, M—male; F—female.

No	Age	Sex	TRMR-LPS	TR-TIP	Additional Symptoms
1	33	F	<60%	<50%	N
2	26	F	<60%	<50%	N
3	21	M	<60%	<50%	N, Sp
4	22	M	<30%	<50%	N, Sp, Sn
5	37	F	<30%	<50%	N, Sp
6	18	M	<5%	<25%	N, Sp

**Table 2:** Patients after surgery.

No	Age	Sex	TRMR-LPS	TR-TIP	Additional Symptoms
1	33	F	>60%	>80%	-
2	26	F	>60%	>80%	-
3	21	M	>60%	>80%	-
4	22	M	>60%	>80%	-
5	37	F	>60%	>80%	-
6	18	M	>60%	>50%	Sp (minor improvement)

## Data Availability

Restrictions apply to the availability of these data. Data was obtained from patients treated at the Department of Cranio-Maxillofacial Surgery, Cracow, Poland, and cannot be shared, in accordance with the General Data Protection Regulation (EU) 2016/679.

## References

[1] Mills N., Pransky S.M., Geddes D.T., Mirjalili S.A. (2019). What is a Tongue Tie? Defining the anatomy of the in-situ lingual frenulum. Clin. Anat..

[2] Mills N., Keough N., Geddes D.T., Pransky S.M., Mirjalili S.A. (2019). Defining the anatomy of the neonatal lingual frenulum. Clin. Anat..

[3] Hill R.R., Lee C.S., Pados B.F. (2020). The prevalence of ankyloglossia in children aged <1 year: A systematic review and meta-analysis. Pediatric Res..

[4] Yousefi J., Tabrizian Namini F., Raisolsadat S.M., Gillies R., Ashkezari A., Meara J.G. (2015). Tongue-tie Repair: Z-Plasty vs. Simple Release. Iran. J. Otorhinolaryngol..

[5] Zaghi S., Shamtoob S., Peterson C., Christianson L., Valcu-Pinkerton S., Peeran Z., Fung B., Kwok-keung Ng D., Jagomägi T., Archambault N. (2021). Assessment of posterior tongue mobility using lingual-palatal suction: Progress towards a functional definition of ankyloglossia. J. Oral Rehabil..

[6] Fuchs G. (1966). Histological examinations of the frenum of the tongue. Dtsch. Stomatol..

[7] Heller J., Gabbay J., O’Hara C., Heller M., Bradley J.P. (2005). Improved Ankyloglossia Correction with Four-Flap Z-Frenuloplasty. Ann. Plast. Surg..

[8] O’Shea J.E., Foster J.P., O’Donnell C.P., Breathnach D., Jacobs S.E., Todd D.A., Davis P.G. (2017). Frenotomy for tongue-tie in newborn infants. Cochrane Database Syst. Rev..

[9] Srinivasan A., Al Khoury A., Puzhko S., Dobrich C., Stern M., Mitnick H., Goldfarb L. (2018). Frenotomy in Infants with Tongue-Tie and Breastfeeding Problems. J. Hum. Lact..

[10] Ghaheri B.A., Cole M., Mace J. (2018). Revision Lingual Frenotomy Improves Patient-Reported Breastfeeding Outcomes: A Prospective Cohort Study. J. Hum. Lact..

[11] Messner A.H., Lalakea M.L. (2000). Ankyloglossia: Controversies in management. Int. J. Pediatric Otorhinolaryngol..

